# Sleep deprivation alters the time course but not magnitude of locomotor sensitization to cocaine

**DOI:** 10.1038/s41598-018-36002-1

**Published:** 2018-12-05

**Authors:** Theresa E. Bjorness, Robert W. Greene

**Affiliations:** 1 Research Service, North Texas VA Health Care System, Dallas, TX 75216 USA; 2 Department of Psychiatry, University of Texas Southwestern, Dallas, TX 75390 USA; 3 Department of Neuroscience, University of Texas Southwestern, Dallas, TX 75390 USA; 4 Research Service, North Texas VA Health Care System, Dallas, TX 75216 USA; 5 International Institute for Integrative Sleep Medicine, University of Tsukuba, Tsukuba, 305-8577 Japan

## Abstract

Repeated exposure to drugs of abuse progressively increases the response to the same stimuli, a process known as sensitization. Behavioral sensitization to cocaine administration is often measured in non-human subjects via locomotor activity which is easily quantifiable. The effects of four hours of sleep deprivation on repeated cocaine (five daily and one challenge) showed attenuated hyperactivity on the first day only, compared to the non-deprived group. Both groups reached the same final level of sensitization, indicating that sleep deprivation altered the time course, but not magnitude of locomotor sensitization.

## Introduction

Sensitization is a progressive increase in the responses to repeated stimuli and is often measured as increased lcomotor activity following repeated administration of drugs of abuse such as cocaine, amphetamine, and morphine^1–5^. While the precise mechanism(s) by which drugs of abuse elicit locomotor sensitization are not entirely clear, there is substantial evidence linking changes in the mesocortical dopaminergic (DA) system to the induction and expression of sensitization^6–9^ including increased DA release within the nucleus accumbens in response to repeated drug administration^10^. Induction of sensitization is dependent on alterations within the ventral tegmental area (VTA)^11–13^ with intra-VTA drug administration sufficient to induce sensitization^14^ and intra-VTA glutamatergic receptor antagonism able to block the development of sensitization^15,16^. Expression of sensitization is dependent on alterations in the broader mesocortical DA system including the nucleus accumbens in which intra-accumbens DA antagonists block expression of sensitization^17^ and, for cocaine, the prefrontal cortex based on evidence from lesion studies^18^ amongst other regions (for review^6^).

Cocaine is a psychostimulant which causes sleep disruption in human users under both chronic use and abstinence conditions^19–21^. Cocaine decreases the amount of time spent in deeper Non-Rapid Eye Movement sleep (NREM, also referred to as slow wave sleep (SWS) stage 3–4) and increases the amount of time spent in lighter stage 2 NREM sleep^22^. Additionally, cocaine reportedly shifts the timing of Rapid Eye Movement sleep (REM) within the sleep cycle to occur earlier within the sleep period^20,22,23^, while overall REM time is increased^22,24^. As with humans, cocaine has been shown to disrupt sleep/waking behavior in rats^25–27^ and we recently demonstrated that acute cocaine dose dependently increases waking in mice with a delayed sleep onset latency of approximately one hour compared to saline administration following a 18 mg/kg dose^28^.

Sleep disruption through deprivation can influence the DA reward system; specifically, short duration (6 h) sleep deprivation decreases neuronal activity in the VTA which lasts beyond the homeostatic recovery period^29^, while longer duration (72 h) sleep deprivation reduces striatal D1 receptor expression although D2 receptor expression is unchanged^30^.

There is also evidence that sleep disruption through deprivation influences the response to drugs such as cocaine. In mice, acute (6 h) sleep deprivation has been shown to increase hyperactivity in response to a single cocaine administration^31^, and increase sensitization to amphetamine as measured by activity to a challenge dose one week following a single induction dose^32^ with sleep deprivation occurring immediately prior to the induction dose. Here, we tested the effect of repeated sleep deprivation in order to determine whether repeated sleep deprivation would alter the magnitude of sensitization to cocaine or induce sensitization in the absence of cocaine.

## Materials and Methods

### Animals

Adult male C57BL/6 mice (age range 2–6 months) were assigned to one of three groups, described below, and placed into cages set atop a treadmill apparatus with food and water available ad libitum. A 12:12-h light/dark cycle was used in rooms with an ambient temperature of 22.0 +/− 1 °C. All experiments were approved by the North Texas VA Health Care System IACUC and were in accordance with recommendations in the Guide for Care and Use of Laboratory Animals (U.S. National Research Council).

### Experimental paradigm

**Figure 1:**
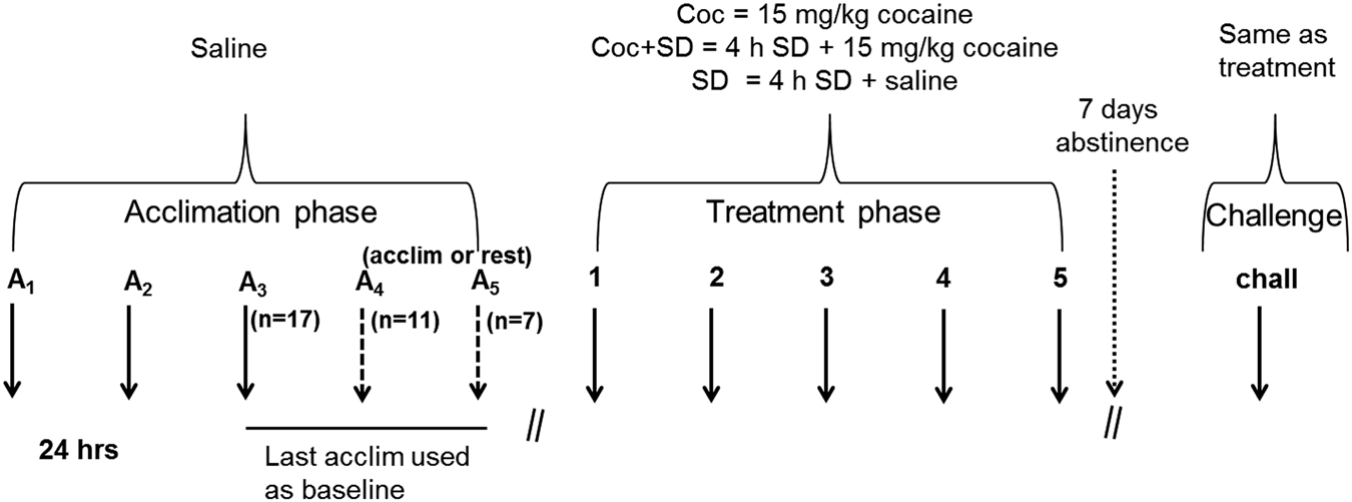
Experimental timeline showing the acclimation (A), treatment (T), and challenge phases along with groups (Coc, Coc + SD, and SD). Mice experienced 3–5 days of acclimation (see methods for description); the number of mice receiving 3, 4, or 5 acclimation days is provided in parentheses. Animals that reached the acclimation criteria prior to the 5 day maximum received 1–2 days of rest prior to the start of the treatment phase.

Mice were injected with saline (IP) and acclimated to a 28 cm × 28 cm open field activity chamber (Med Associates, Activity Monitor SOF-812) for 1 h per day between ZT 3.5–7 for at least 3 days (Fig. 1). Mice were given up to 5 acclimation sessions after which they were excluded if they did not show a reduction in activity from the first to last acclimation day and second-to-last to last acclimation day (i.e. decrease in activity from the initial day along with a pattern of two days of decreased activity prior to the start of the cocaine phase). This relatively extensive acclimation was used to encourage full habituation prior to any sleep deprivation since sleep deprivation can increase activity in a novel environment^33^. There was no difference in acclimation days between groups (all averaged 3.7 days). Next, mice underwent a 5-day treatment phase consisting of daily cocaine (15 mg/kg, IP) or saline injected immediately prior to the 1-hour activity session. One group of mice was undisturbed prior to receiving cocaine (Coc, n = 12), another group of mice was sleep deprived for 4 h immediately prior to receiving cocaine (Coc + SD, n = 13), and a third group was sleep deprived for 4 h immediately prior to receiving saline (SD, n = 10). Sleep deprivation was achieved via the treadmill method (forced slow walking using bottomless cages set atop a treadmill belt)^34^. After a one-week abstinence period, mice were given a challenge (Chall) cocaine (15 mg/kg) injection and activity was monitored for 1 h. As during the treatment phase, mice in the Coc + SD and SD groups were sleep deprived for 4 h immediately prior to the challenge. All testing occurred under dim red light conditions between ZT 3.5–7. The cocaine dose used (15 mg/kg) is within the typical range used for locomotor sensitization experiments in rodents^35^.

### Outcome measurements

Beam breaks, distance moved (in cm, defined as consecutive beam breaks with a 0.5 sec resting delay threshold that would end the ambulatory episode), and stereotypic counts (beam breaks within a small area, 6.35 × 6.35 cm) were the main outcome measures. Activity (beam breaks and distance values) across the acclimation through challenge protocol was compared using a Two-way repeated measures ANOVA with Tukey corrections for multiple comparisons. Percent change from the last day of the acclimation phase to the first day of the daily treatment phase (effect of sleep deprivation on response to initial cocaine exposure) and from the last day of the acclimation phase to the challenge day (effect of sleep deprivation on sensitization) was compared using a One-way ANOVA with Holm-Sidak correction for multiple comparisons. For comparison to a theoretical mean of 0, a One-sample t test was used. Additionally, daily percent change across acclimation through challenge was performed using a Two-way repeated measures ANOVA with Tukey corrections for multiple comparisons. For activity in 5-min bins within each session, a Two-way repeated measures ANOVA with Tukey corrections for multiple comparisons was used to compare groups each day. Activity on treatment day 1 was compared using a One-way ANOVA with Holm-Sidak correction for multiple comparisons and the distributions of activity were compared using a Kolmogorov-Smirnov test, while the normality of the distributions of activity were determined using a Kolmogorov-Smirnov normality test. Correlation between activity on the first treatment day and challenge day was performed using Pearson [Coc, SD] and Spearman [Coc + SD, failed normality test] correlations. Time bin of peak activity (1 h activity period divided into 3, 20 min bins) was compared using a Two-way repeated measures ANOVA with Tukey corrections for multiple comparisons. Additionally, average speed (cm/s) and number of bins with average of 0 speed (used as a measure of quiescence) was compared using a Two-way repeated measures ANOVA with Tukey corrections for multiple comparisons. One mouse was excluded based on extremely low activity during the acclimation phase (identified as an outlier, via ROUT method). All statistical analyses were performed using GraphPad Prism and all tests were two tailed. The significance level was set at *p* < 0.05 with actual P values given whenever possible and the values provided are mean +/−  standard error. Data is available upon request.

## Results

### SD decreased the locomotor response to initial cocaine, but did not alter sensitization to repeated cocaine

**Figure 2:**
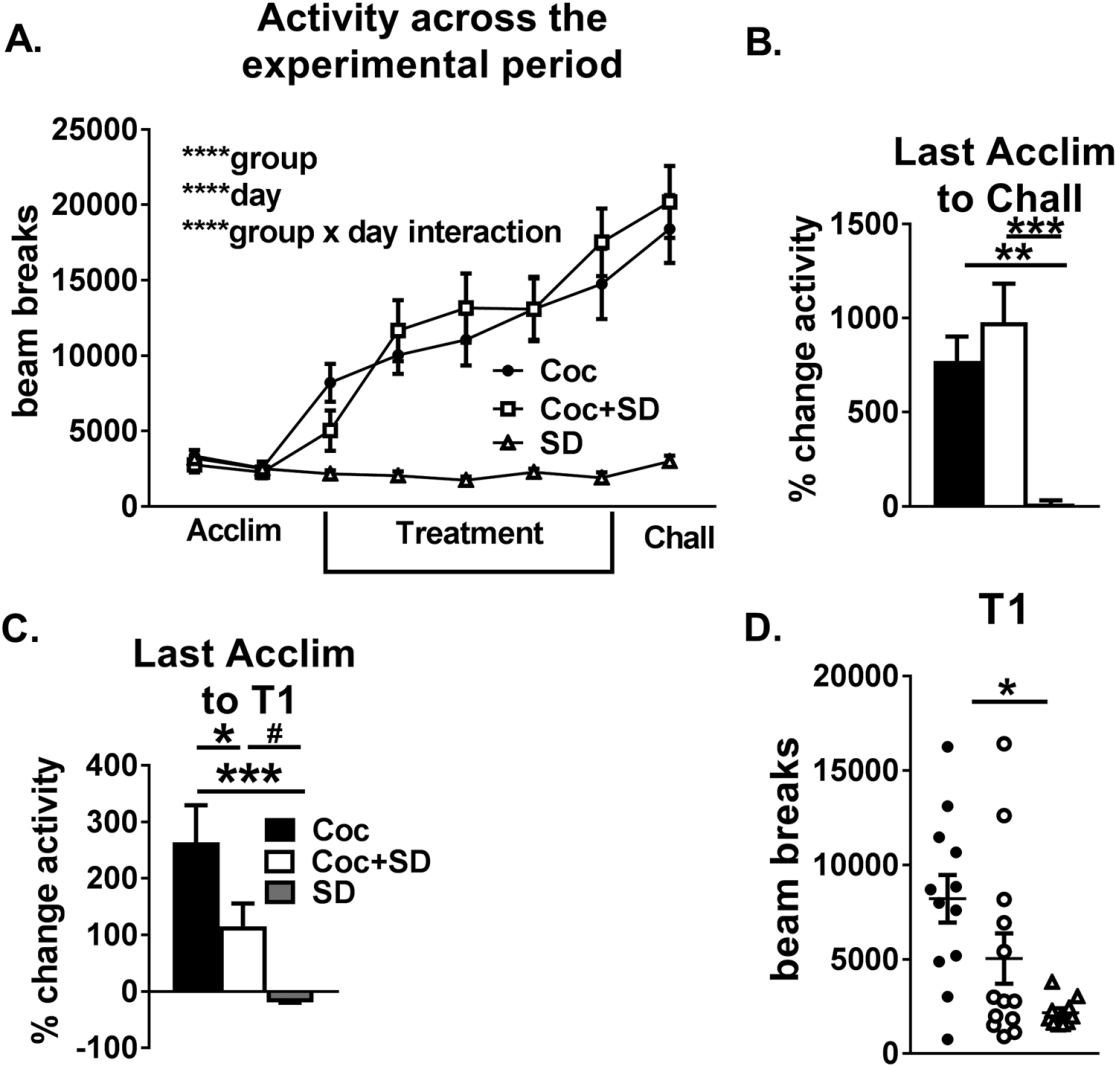
Sleep deprivation initially reduces than subsequently increases the locomotor response to cocaine without altering the magnitude of sensitization. (**A**) Coc and Coc + SD groups showed similar patterns of absolute activity throughout the acclimation to treatment to challenge phases, while the SD group showed relatively slight (but increasing) activity from the first to last SD days. (**B**) Coc + SD and Coc groups showed similar levels of sensitization as determined by the increase in activity from the last acclimation day to the challenge day, while the SD group showed no significant increase. (**C**) Sleep deprivation reduced the acute response to cocaine as determined by the percent change beam breaks from the last acclimation day to the first treatment day in the Coc + SD group compared to the Coc group (**D**). In the Coc group absolute activity in response to the initial cocaine was distributed in a rough bell shaped pattern, while activity in Coc + SD and SD groups was more clustered towards the low end of activity. All values are expressed as mean +/− SEM, n = 10–13/group, ^*#*^*p* < *0.1*, *p < *0.05*, **p < *0.005*, ***p < *0.0005*, ****p < *0.0001*, and bars denote which groups differ.

All groups showed a similar decrease in activity across the acclimation phase (One-way ANOVA Coc −33.8 +/− 6.3%, Coc + SD −25.2 +/− 4.5%, SD −24.8 +/− 4.3%, *p* = *0.97*, data not shown). For absolute activity values measured as beam breaks, the Coc and Coc + SD groups showed a similar increasing pattern throughout the treatment and challenge phases, while the SD group showed little change across days with group differences attributable to the SD group (Two-way ANOVA, group *p* < *0.0001*, day *p* < *0.0001*, group × day interaction *p* < *0.0001*, Fig. 2A). Coc and Coc + SD groups reached a similar increase in total activity in response to cocaine as determined by the change in activity from the last acclimation day through the challenge phase (One-way ANOVA, Coc vs Coc + SD *p* = *0.6*, Coc vs SD *p* = *0.005*, Coc + SD vs SD *p* = *0.0003*, Fig. 2B), while the SD group did not show an overall increase in activity from the last acclimation day through the challenge phase (One sample T test Coc *p* < *0.0001*, Coc + SD *p* = *0.0004*, SD *p* = *0.4*). All Coc and Coc + SD animals met the criteria for sensitization using a threshold of >20% increase in activity from the first treatment day to the challenge day^4^, as did a subset of SD animals (60%). Mice that were sleep deprived immediately prior to receiving cocaine showed less initial activity in response to cocaine than the non-deprived animals as determined by the percent increase in activity from the last acclimation trial to the first cocaine trial, while they showed a non-significant increase in activity compared to sleep deprived mice that received saline (One-way ANOVA Coc vs Coc + SD *p* = *0.05*, Coc + SD vs SD *p* = *0.07*, Fig. 2C). As expected, non-deprived mice that received cocaine showed a greater increase in activity than sleep deprived mice that received saline (One-way ANOVA Coc vs SD *p* = *0.001)*. Both cocaine exposed groups showed increases in activity from the last acclimation trial to the first cocaine trial that were significantly greater than 0 (One sample T test Coc *p* = *0.002*, Coc + SD *p* = *0.013*), while the non-cocaine exposed group did not (SD *p* = *0.18*). On the first treatment day, there was a group difference in absolute activity that was primarily driven by differences between Coc and SD groups (One-way ANOVA Coc vs Coc + SD *p* = *0.14*, Coc vs SD *p* = *0.003*, Coc + SD vs SD *p* = *0.25*, Fig. 2D). There was also a similar spread of activity within the Coc and Coc + SD groups, however, while activity in the Coc animals was normally distributed throughout the activity range (Kolmogrov-Smirnov normality test, *p* > *0.1*), Coc + SD animals showed a relatively high proportion of animals bunched toward the low end of the activity range (Kolmogrov-Smirnov normality test, *p* = *0.007*). The distributions of the Coc and SD groups were significantly different from each other, while Coc + SD was not significantly different than either, although the comparison with Coc reached a trend towards significance (Kolmogorov-Smirnov test, Coc vs SD *p* = *0.001*, Coc vs Coc + SD *p* = *0.058*, Coc + SD vs SD *p* = *0.28*). Furthermore, there was a significant correlation between activity in response to the first treatment day and the challenge day in the Coc group, but not in Coc + SD or SD groups (Pearson correlation, Coc R^2^ = 0.61, *p* = *0.003*, SD R^2^ = 0.12, *p* = *0.34*, Spearman correlation, Coc + SD R^2^ = 0.12, *p* = *0.25*]). The differences in the timing of activity changes between sleep deprived and non-sleep deprived cocaine treated groups become apparent when comparing daily increases in activity. The Coc + SD group showed large increases in activity on both the first and second treatment days, while the Coc group showed large increases in activity only on the first treatment day and the SD group showed decreases in activity until the fourth treatment day (Two-way ANOVA, group *p* = *0.0013*, day *p* < *0.0001*, group*day interaction *p* < *0.0001*, Supplementary Fig. 1). Overall, this suggests that sleep deprivation prior to repeated cocaine does not influence the magnitude of the response to cocaine, but does alter the time course of activity across days.

### Similar pattern of activity within treatment sessions

**Figure 3:**
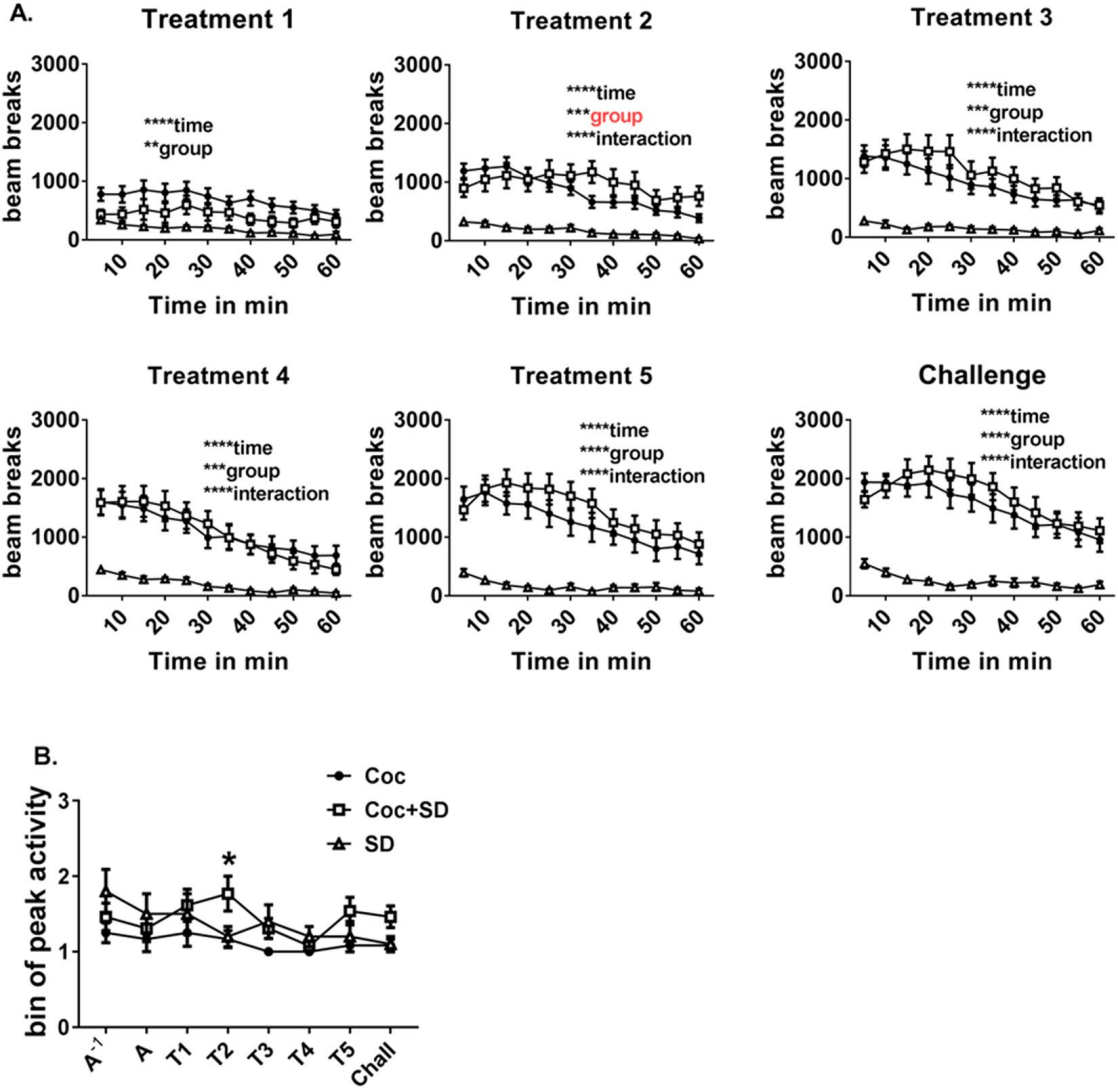
Sleep deprivation alters the within-session pattern of activity following the second cocaine exposure. (**A**) All groups showed a similar pattern of activity within the 1 h post-injection session of high initial activity tapering across the period except for a flatter pattern of activity in the Coc + SD groups on the second treatment day. (**B**) This flattened pattern of activity is evident as a delayed peak in activity within the post-injection period. All values are expressed as mean +/− SEM, n = 10–13/group, *p < *0.05*, **p < *0.005*, ***p < *0.0005*, ****p < *0.0001*.

The typical pattern of activity was initially high, followed by a steady reduction across the 1 h period (Two-way ANOVA, significant effect of time all days, *p* < *0.0001*) with higher activity in the Coc and SD + Coc groups compared to the SD group (group effect *p* = *0.0043* [T1], *p* = *0.0003* [T2, 3, 4], *p* < *0.0001* [T5, Chall], group*time interaction *p* < *0.0001*); however, the Coc + SD group showed significantly higher activity than the Coc group during the middle portion of treatment day 2 (*p* < *0.05)* which may account for the increased relative activity (% change across time detailed above) in this group on this day (Fig. 3A). In order to further investigate the pattern of activity within the 1 h post-injection sessions, each session was divided into 3, 20 min bins with a numerical value assigned to the bin in which the peak activity occurred (for example, if the peak activity of the session occurred in the first 20 min, the session would be assigned a value of 1, while an activity peak occurring in the second or third 20 min bin would be assigned 2 or 3, respectively). When bin of peak activity across days was compared between groups, peak activity occurred later in the Coc + SD group on treatment day 2 compared to Coc and SD groups (Two-way ANOVA, day *p* = *0.047*, group *p* = *0.01*, Fig. 3B).

### Increased in beam breaks reflect increased distance moved and stereotypic activity

**Figure 4:**
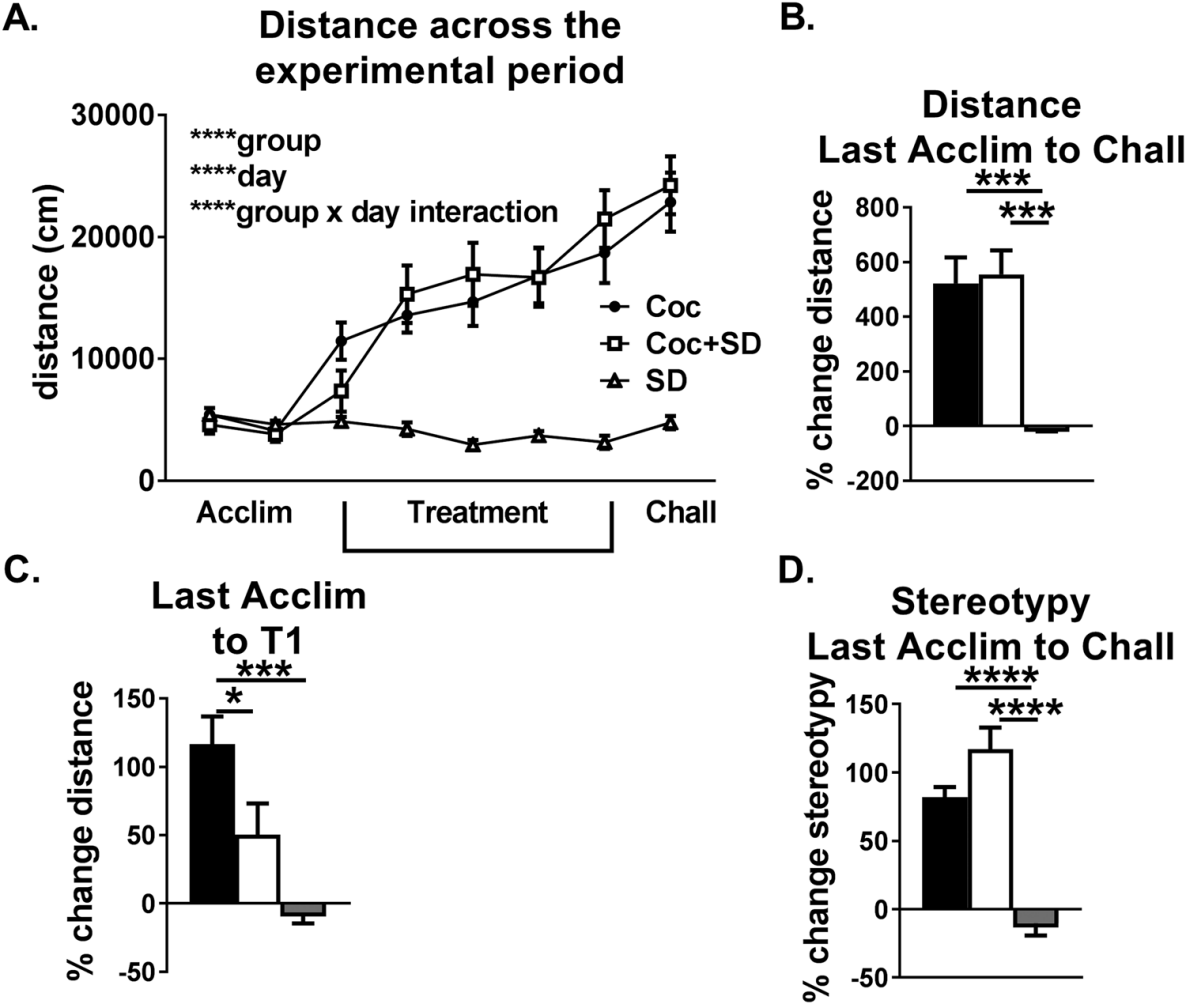
Effect of sleep deprivation replicated with distance and stereotypy as outcomes. (**A**) For absolute distance across the experimental period, the Coc and Coc + SD groups show a similar pattern of increasing distance, while SD animals are fairly stable similar to the beam breaks across time. (**B,C**) The similarity in sensitization between Coc and Coc + SD groups was replicated with distance (**B**), as was the reduced initial response to cocaine (**C**) although there were differences in significance levels for some of the group comparisons. (**D**) Finally, sensitization as measured by stereotypic counts was also similar between Coc and Coc + SD groups. (**D**) All values are expressed as mean +/− SEM, n = 10–13/group, ^*#*^*p* < *0.1*, ***p < *0.0005*, ****p < *0.0001*, and bars denote which groups differ.

The pattern of activity seen with beam breaks between groups is mostly recapitulated when using distance (cm) as the outcome measure (Fig. 4A–C). The pattern of increasing activity across treatment and challenge days was similar between Coc and Coc + SD animals (Two-way ANOVA, day *p* < *0.0001*, group *p* < *0.0001*, day*group interaction *p* = *0.0001*, no significant difference between Coc and Coc + SD via Tukey’s multiple comparison test, Fig. 4A). As with activity measured in beam breaks, both distance moved and stereotypic counts were similarly increased from acclimation to the challenge day in Coc and Coc + SD groups, while significantly lower in the SD group (distance, One-way ANOVA Coc vs Coc + SD *p* = *0.98*, Coc vs SD *p* = *0.0004*, Coc + SD vs SD *p* = *0.0001*, stereotypic counts, One-way ANOVA Coc vs Coc + SD *p* = *0.12*, Coc vs SD *p* < *0.0001*, Coc + SD vs SD *p* < *0.0001*, Fig. 4B,D). The reduced initial response to cocaine measured with beam breaks was apparent with distance although the significance level was reduced. When comparing increase in activity as measured by distance from the last acclimation to first treatment trial there was a significant difference between Coc and SD groups (One-way ANOVA, *p* = *0.004)* and Coc + SD vs Coc groups (*p* = *0.05)*, while the trend between Coc + SD vs SD was lost (*p* = *0.14*, Fig. 4C).

### Sleep deprivation did not influence movement speed

**Figure 5:**
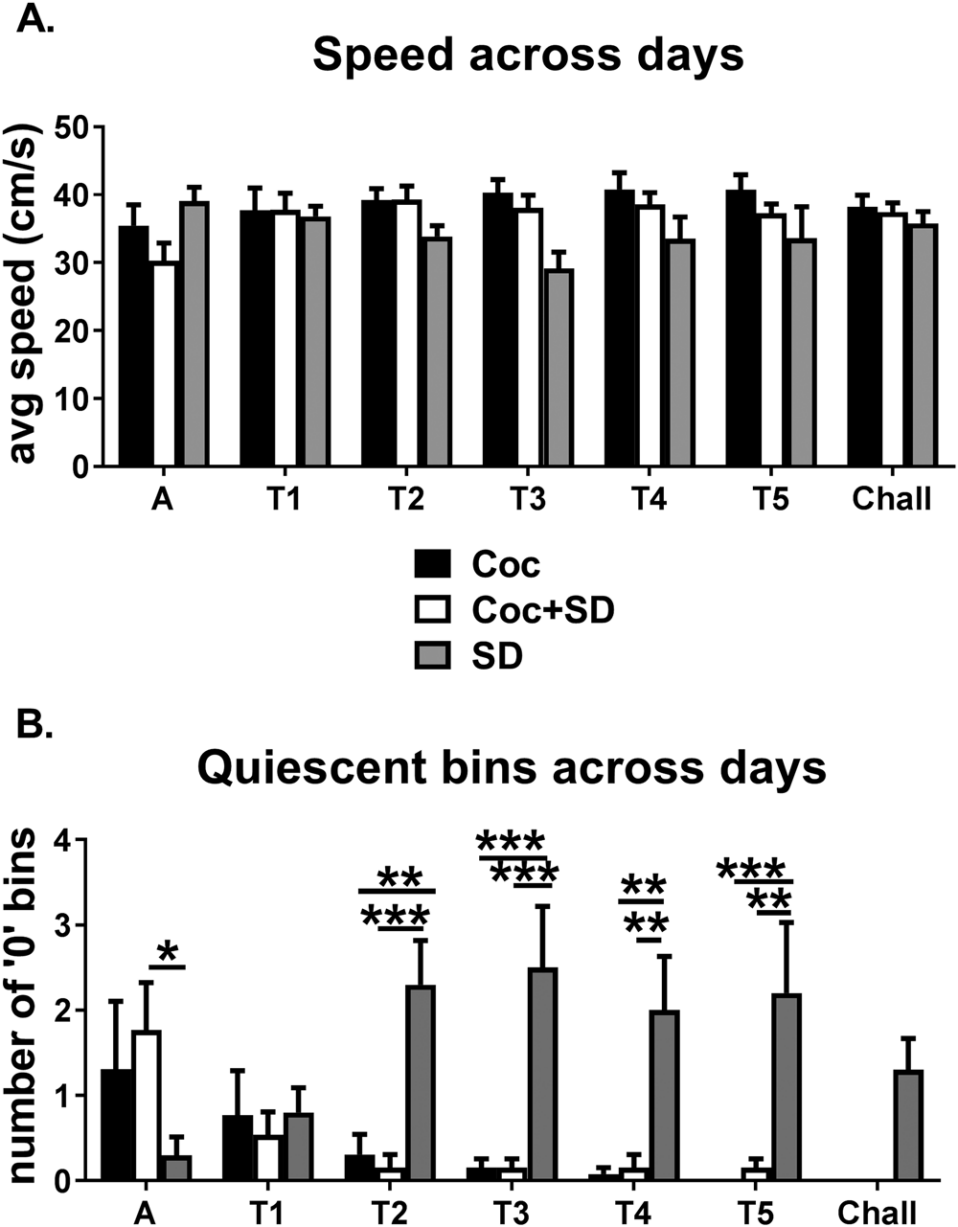
Sleep deprivation did not influence locomotor activity speed. (**A**) There was no difference in speed between groups from acclimation through challenge. (**B**) SD animals showed a higher number of bins with 0 average movement speed on several of the treatment days compared to both Coc and Coc + SD animals. All values are expressed as mean +/− SEM, n = 10–13/group, *p < *0.05*, **p < *0.005*, ***p < *0.0005*, and bars denote which groups differ.

Across the acclimation, treatment, and challenge phases, there was no overall difference in movement speed between groups; however, there was a significant group × day interaction due to slower movement in the SD group on some days (Two-way ANOVA, group *p* = *0.22*, day *p* = *0.39*, group × day interaction *p* < *0.005*, Fig. 5A). Furthermore, the number of 5 min bins/day with an average movement speed of 0 (quiescence as a rough indicator of possible sleeping within the activity chamber) was significantly different between groups, which again was driven by the SD group (Two-way ANOVA, group *p* < *0.005*, day *p* = *0.34*, group × day interaction *p* < *0.001*, Fig. 5B) suggesting that the reduced locomotor response to cocaine on treatment day 1 in the Coc + SD group compared to the Coc group was likely not due to Coc + SD animals sleeping within the activity chamber.

## Discussion

Acute daily SD did not influence overall locomotor sensitization to cocaine with the hypoactivity in response to the initial cocaine administration overcome by increases in activity to subsequent cocaine administrations. The reduced activity response following the initial cocaine exposure in Coc + SD animals was unexpected given the prior finding of SD increasing hyperactivity in response to cocaine^31^. However, the discrepancy in results may be due to methodological differences, with Andersen and colleagues using much shorter sessions for habituation (10 min/d for 3 days) and not including IP injections within the habituation procedure. If mice were not fully habituated to the testing environment, SD could enhance activity since SD enhances preference to novelty^36^. While it is not possible to entirely exclude the possibility that Coc + SD animals fell asleep in the activity chamber, there was no significant difference between Coc vs Coc + SD animals when comparing the number of 5 min bins with a movement speed of average 0 cm/s on any day including the first treatment day, whereas the SD group showed significantly more 0 movement bins on several days. Furthermore, we previously found that following a similar dose of cocaine (18 mg/kg), latency to fall sleep was ~80 min^28^. Together, this suggests that the decreased initial response to cocaine in the Coc + SD group was likely not due to Coc + SD animals sleeping within the 1 h post cocaine period. Importantly, the method of sleep deprivation used (forced slow walking) was not expected to induce fatigue since belt speed needed to prevent sleep (~3 cm/s) is considerably slower than speeds used for treadmill exercise in mice (~20 cm/s)^37^.

The unusual pattern of activity in which activity peaked during the middle portion of the 1 h post-injection period in Coc + SD animals on the second treatment day was also unanticipated; however, instances of peak activity occurring later within a post-cocaine administration period has been demonstrated following repeated cocaine exposure^1^.

The lack of change in sensitization in the Coc + SD group appears to be driven by both the lower response to the initial cocaine administration along with the relatively high response to the second cocaine administration (Supplementary Fig. S1). Following the initial cocaine administration, Coc animals showed a wide range in activity, while Coc + SD animals were bunched towards the low end of the activity range. It has previously been demonstrated that animals with high initial response to cocaine show lower sensitization^38,39^; accordingly, there was a significant correlation between response to the initial cocaine exposure and challenge exposure in the Coc group but not in the Coc + SD group. The differences in initial activity were insufficient to translate into differences in sensitization which may also be influenced by sleep deprivation itself since daily acute sleep deprivation in the absence of cocaine induced sensitization in a subset of animals although there was no group effect (i.e. percent change activity from last acclimation through challenge was not significantly greater than 0). Increases in activity following sleep deprivation have been demonstrated previously in that sleep deprivation (12 h by rotating drum) can reverse defeat-induced activity reductions^40^ and prolonged sleep deprivation targeting REM sleep deprivation is a model for mania^41^, based in part on hyperactivity following deprivation^42,43^ which is associated with increased D1 receptor expression in the limbic system^44^, while others have shown widespread increased D2 receptor expression following prolonged REM sleep deprivation^45^.

The mechanism by which sleep deprivation can influence the development of behavioral sensitization to a psychostimulant is an open question but appears to be primarily due to the comparative hypoactivity upon the initial cocaine administration. Given that animals sleep deprived in the absence of cocaine also showed a decrease in activity following the initial sleep deprivation exposure, this suggests that increased sleep drive, even if sleep itself is not achieved, is sufficient to attenuate the arousal promoting effects of cocaine. This attenuation is transient in that upon the second cocaine exposure there is a strong increase in locomotor activity following sleep deprivation. Interestingly, sleep deprivation alone resulted in sensitization in a subset of animals indicating that there may be complex interactions between sleep drive, which is expected to be increased following sleep deprivation, and arousal, which is expected to be increased in the presence of a conditioned procedure and environment (conditioning in the sense that animals were removed from their home cage in one room, weighed, transported into a different room with different lighting conditions, given an IP injection, and placed in a large square cage recently wiped with a germicidal cloth). Cocaine causes psychostimulation by blockade of the DA, serotonin, and norephinephrine transporters^46,47^ such that higher arousal at the time of cocaine exposure, and therefore higher DA levels, would result in greater overall extracellular DA, thereby facilitating increased motor activity. Cocaine is also reinforcing based on its blockade of the DA transporter^48^ which may explain the transient nature of the reduced motor response in sleep deprived mice. Since the activity box would likely become associated with receipt of a reinforcing stimulus, it would not be unexpected for a shift of the sleep drive/arousal spectrum towards arousal to occur. The mechanism by which sleep deprivation alone induces sensitization in a subset of animals is less clear; however, it is important to note that the increase in activity across the sleep deprivation phase was substantially less than the cocaine exposed groups suggesting that this sensitization is quite modest. Additionally, it has previously been demonstrated that repeated saline administrations can increase amphetamine-induced DA release^49^.

Alternatively, it is possible that animals became habituated to repeated sleep deprivation as has been demonstrated by diminishing slow wave activity (SWA) power rebound under chronic sleep deprivation conditions^50,51^, although stable rebound across multiple days of chronic sleep deprivation has also been demonstrated^52^. Under the conditions of the present study, it is unlikely that animals in the current experiment became habituated to the repeated sleep deprivation since SWA power rebound is similar across 7 days of 4 h acute sleep deprivation by the treadmill method that we employed (Supplementary Fig. S2). Overall, this argues that the transient nature of the effect of sleep deprivation is not due to changes in response to sleep deprivation over time, but may be due to changes in the effect of sleep deprivation on cocaine response over time (i.e. possible interaction between arousal and sleep drive discussed above). However, sleep drive (as determined by SWA rebound) cannot necessarily be equated with changes in motor activity as a consequence of sleep deprivation.

One limitation to the current experiment is the use of a single acute injection and challenge protocol. It has been shown that sensitization to a challenge amphetamine administration varies based on the challenge dose and time from the previous injection with tolerance following low dose challenge early in withdrawal^53^. Thus, it is possible that the effect of SD could vary by cocaine exposure protocol. Additionally, the stereotypy measure was based on the beam breaks within a small area which may miss more subtle movements (detection threshold ~1.5 cm) such as head movements which have been shown to increase following repeated cocaine^54^. There was no control group of repeated saline without sleep deprivation. However, all mice experienced this condition during the acclimation phase and showed a decrease in activity during this period suggesting that further saline administration would likely not induce sensitization. Neural activity during the sleep deprivation period was not measured; however, the treadmill method of sleep deprivation has been verified to reliably prevent sleep over the course of a 4 h period^28,34^ such that it is unlikely that sleep deprived mice achieved sleep and it is unlikely that the non-deprived mice (Coc) were spontaneously awake for a majority of the same 4 h period given that sleep drive peaks early in the light phase^55^.

In sum, acute sleep deprivation modestly influenced the development of locomotor sensitization to cocaine, primarily by decreasing the initial response to cocaine. This suggests that the development of the activity based drug response may be transiently delayed, though ultimately unchanged in magnitude, with sleep deprivation. Future experiments are needed to investigate the mechanism(s) by which sleep loss alters the time course of behavioral sensitization to cocaine and whether this subtle alteration in sensitization is associated with alterations in the development of other addiction-related behaviors such as reward seeking. Currently, there is indirect evidence that sleep disruption may increase relapse to cocaine taking in humans^56^, while circadian distributed sleep disruption increases effort to obtain cocaine in a subset of animals as measured by progressive ratio responding for self-administered cocaine^57^. Recently, it was shown that sleep deprivation induces conditioned place preference to amphetamine using a protocol that did not induce preference in non-deprived controls^58^. Finally, additional experiments are needed to determine whether the effect of sleep deprivation on the development of locomotor sensitization would generalize to non-stimulant drugs such as morphine.

## Electronic supplementary material

Supplementary information

## References

[1] Post RM, Rose H (1976). Increasing effects of repetitive cocaine administration in the rat. Nature.

[2] Robinson TE, Becker JB (1986). Enduring changes in brain and behavior produced by chronic amphetamine administration: a review and evaluation of animal models of amphetamine psychosis. Brain research.

[3] Henry DJ, White FJ (1995). The persistence of behavioral sensitization to cocaine parallels enhanced inhibition of nucleus accumbens neurons. The Journal of neuroscience: the official journal of the Society for Neuroscience.

[4] Pierce RC, Bell K, Duffy P, Kalivas PW (1996). Repeated cocaine augments excitatory amino acid transmission in the nucleus accumbens only in rats having developed behavioral sensitization. The Journal of neuroscience: the official journal of the Society for Neuroscience.

[5] Robinson TE, Browman KE, Crombag HS, Badiani A (1998). Modulation of the induction or expression of psychostimulant sensitization by the circumstances surrounding drug administration. Neuroscience and biobehavioral reviews.

[6] Pierce RC, Kalivas PW (1997). A circuitry model of the expression of behavioral sensitization to amphetamine-like psychostimulants. Brain research. Brain research reviews.

[7] Wolf ME (1998). The role of excitatory amino acids in behavioral sensitization to psychomotor stimulants. Progress in neurobiology.

[8] Vanderschuren LJ, Kalivas PW (2000). Alterations in dopaminergic and glutamatergic transmission in the induction and expression of behavioral sensitization: a critical review of preclinical studies. Psychopharmacology.

[9] Thomas MJ, Kalivas PW, Shaham Y (2008). Neuroplasticity in the mesolimbic dopamine system and cocaine addiction. British journal of pharmacology.

[10] Robinson TE, Jurson PA, Bennett JA, Bentgen KM (1988). Persistent sensitization of dopamine neurotransmission in ventral striatum (nucleus accumbens) produced by prior experience with (+)-amphetamine: a microdialysis study in freely moving rats. Brain research.

[11] Carlezon WA, Nestler EJ (2002). Elevated levels of GluR1 in the midbrain: a trigger for sensitization to drugs of abuse?. Trends in neurosciences.

[12] Kauer JA (2004). Learning mechanisms in addiction: synaptic plasticity in the ventral tegmental area as a result of exposure to drugs of abuse. Annual review of physiology.

[13] Borgland SL, Taha SA, Sarti F, Fields HL, Bonci A (2006). Orexin A in the VTA is critical for the induction of synaptic plasticity and behavioral sensitization to cocaine. Neuron.

[14] Kalivas PW, Weber B (1988). Amphetamine injection into the ventral mesencephalon sensitizes rats to peripheral amphetamine and cocaine. The Journal of pharmacology and experimental therapeutics.

[15] Vezina P, Queen AL (2000). Induction of locomotor sensitization by amphetamine requires the activation of NMDA receptors in the rat ventral tegmental area. Psychopharmacology.

[16] Licata SC, Schmidt HD, Pierce RC (2004). Suppressing calcium/calmodulin-dependent protein kinase II activity in the ventral tegmental area enhances the acute behavioural response to cocaine but attenuates the initiation of cocaine-induced behavioural sensitization in rats. The European journal of neuroscience.

[17] Abrahao KP, Quadros IM, Souza-Formigoni ML (2011). Nucleus accumbens dopamine D(1) receptors regulate the expression of ethanol-induced behavioural sensitization. The international journal of neuropsychopharmacology.

[18] Pierce RC, Reeder DC, Hicks J, Morgan ZR, Kalivas PW (1998). Ibotenic acid lesions of the dorsal prefrontal cortex disrupt the expression of behavioral sensitization to cocaine. Neuroscience.

[19] Coffey SF, Dansky BS, Carrigan MH, Brady KT (2000). Acute and protracted cocaine abstinence in an outpatient population: a prospective study of mood, sleep and withdrawal symptoms. Drug Alcohol Depend..

[20] Pace-Schott EF (2005). Sleep quality deteriorates over a binge–abstinence cycle in chronic smoked cocaine users. Psychopharmacology (Berl).

[21] Angarita GA, Emadi N, Hodges S, Morgan PT (2016). Sleep abnormalities associated with alcohol, cannabis, cocaine, and opiate use: a comprehensive review. Addict Sci Clin Pract.

[22] Irwin MR, Bjurstrom MF, Olmstead R (2016). Polysomnographic measures of sleep in cocaine dependence and alcohol dependence: Implications for age-related loss of slow wave, stage 3 sleep. Addiction.

[23] Johanson CE, Roehrs T, Schuh K, Warbasse L (1999). The effects of cocaine on mood and sleep in cocaine-dependent males. Experimental and clinical psychopharmacology.

[24] Valladares EM, Irwin MR (2007). Polysomnographic sleep dysregulation in cocaine dependence. ScientificWorldJournal.

[25] Chen B (2015). Sleep Regulates Incubation of Cocaine Craving. J. Neurosci..

[26] Dugovic C, Meert TF, Ashton D, Clincke GH (1992). Effects of ritanserin and chlordiazepoxide on sleep-wakefulness alterations in rats following chronic cocaine treatment. Psychopharmacology (Berl).

[27] Knapp CM, Datta S, Ciraulo DA, Kornetsky C (2007). Effects of low dose cocaine on REM sleep in the freely moving rat. Sleep Biol Rhythms.

[28] Bjorness, T. E. & Greene, R. W. Dose response of acute cocaine on sleep/waking behavior in mice. *Neurobiology of Sleep and Circadian Rhythms* In Press (2018). 10.1016/j.nbscr.2018.02.001 PMC6584645 31236515

[29] Fifel K, Meijer JH, Deboer T (2018). Circadian and Homeostatic Modulation of Multi-Unit Activity in Midbrain Dopaminergic Structures. Scientific reports.

[30] Lim MM, Xu J, Holtzman DM, Mach RH (2011). Sleep deprivation differentially affects dopamine receptor subtypes in mouse striatum. Neuroreport.

[31] Berro LF (2014). Acute total sleep deprivation potentiates cocaine-induced hyperlocomotion in mice. Neuroscience letters.

[32] Saito LP (2014). Acute total sleep deprivation potentiates amphetamine-induced locomotor-stimulant effects and behavioral sensitization in mice. Pharmacology, biochemistry, and behavior.

[33] van Hulzen ZJ, Coenen AM (1981). Paradoxical sleep deprivation and locomotor activity in rats. Physiology & behavior.

[34] Bjorness TE, Kelly CL, Gao T, Poffenberger V, Greene RW (2009). Control and function of the homeostatic sleep response by adenosine A1 receptors. J. Neurosci..

[35] Smith LN (2014). Fragile X mental retardation protein regulates synaptic and behavioral plasticity to repeated cocaine administration. Neuron.

[36] Moore JD, Hayes C, Hicks RA (1979). REM sleep deprivation increases preference for novelty in rats. Physiology & behavior.

[37] Um HS (2011). Treadmill exercise represses neuronal cell death in an aged transgenic mouse model of Alzheimer’s disease. Neurosci Res.

[38] Sabeti J, Gerhardt GA, Zahniser NR (2003). Individual differences in cocaine-induced locomotor sensitization in low and high cocaine locomotor-responding rats are associated with differential inhibition of dopamine clearance in nucleus accumbens. The Journal of pharmacology and experimental therapeutics.

[39] Yamamoto DJ (2013). Rats classified as low or high cocaine locomotor responders: a unique model involving striatal dopamine transporters that predicts cocaine addiction-like behaviors. Neuroscience and biobehavioral reviews.

[40] Meerlo P, Overkamp GJ, Benning MA, Koolhaas JM, Van den Hoofdakker RH (1996). Long-term changes in open field behaviour following a single social defeat in rats can be reversed by sleep deprivation. Physiology & behavior.

[41] Gessa GL, Pani L, Fadda P, Fratta W (1995). Sleep deprivation in the rat: an animal model of mania. European neuropsychopharmacology: the journal of the European College of Neuropsychopharmacology.

[42] Martinez-Gonzalez D (2004). REM sleep deprivation induces changes in coping responses that are not reversed by amphetamine. Sleep.

[43] Valvassori SS (2017). Lithium ameliorates sleep deprivation-induced mania-like behavior, hypothalamic-pituitary-adrenal (HPA) axis alterations, oxidative stress and elevations of cytokine concentrations in the brain and serum of mice. Bipolar disorders.

[44] Demontis MG, Fadda P, Devoto P, Martellotta MC, Fratta W (1990). Sleep deprivation increases dopamine D1 receptor antagonist [3H]SCH 23390 binding and dopamine-stimulated adenylate cyclase in the rat limbic system. Neuroscience letters.

[45] Nunes Junior GP, Tufik S, Nobrega JN (1994). Autoradiographic analysis of D1 and D2 dopaminergic receptors in rat brain after paradoxical sleep deprivation. Brain research bulletin.

[46] Komiskey HL, Miller DD, LaPidus JB, Patil PN (1977). The isomers of cocaine and tropacocaine: effect on 3H-catecholamine uptake by rat brain synaptosomes. Life sciences.

[47] Knapp S, Mandell AJ (1972). Narcotic drugs: effects on the serotonin biosynthetic systems of the brain. Science.

[48] Ritz MC, Lamb RJ, Goldberg SR, Kuhar MJ (1987). Cocaine receptors on dopamine transporters are related to self-administration of cocaine. Science.

[49] Wilcox RA, Robinson TE, Becker JB (1986). Enduring enhancement in amphetamine-stimulated striatal dopamine release *in vitro* produced by prior exposure to amphetamine or stress *in vivo*. Eur. J. Pharmacol..

[50] Clasadonte J, McIver SR, Schmitt LI, Halassa MM, Haydon PG (2014). Chronic sleep restriction disrupts sleep homeostasis and behavioral sensitivity to alcohol by reducing the extracellular accumulation of adenosine. J. Neurosci..

[51] Kim Y (2012). Decoupling of sleepiness from sleep time and intensity during chronic sleep restriction: evidence for a role of the adenosine system. Sleep.

[52] Leemburg S (2010). Sleep homeostasis in the rat is preserved during chronic sleep restriction. Proceedings of the National Academy of Sciences of the United States of America.

[53] Bickerdike MJ, Abercrombie ED (1997). Striatal acetylcholine release correlates with behavioral sensitization in rats withdrawn from chronic amphetamine. The Journal of pharmacology and experimental therapeutics.

[54] Flagel SB, Robinson TE (2007). Quantifying the psychomotor activating effects of cocaine in the rat. Behavioural pharmacology.

[55] Trachsel L, Tobler I, Borbely AA (1986). Sleep regulation in rats: effects of sleep deprivation, light, and circadian phase. The American journal of physiology.

[56] Angarita GA, Canavan SV, Forselius E, Bessette A, Morgan PT (2014). Correlates of polysomnographic sleep changes in cocaine dependence: self-administration and clinical outcomes. Drug and alcohol dependence.

[57] Puhl MD, Boisvert M, Guan Z, Fang J, Grigson PS (2013). A novel model of chronic sleep restriction reveals an increase in the perceived incentive reward value of cocaine in high drug-taking rats. Pharmacol. Biochem. Behav..

[58] Berro LF, Tufik SB, Frussa-Filho R, Andersen ML, Tufik S (2018). Sleep deprivation precipitates the development of amphetamine-induced conditioned place preference in rats. Neurosci. Lett..

