# Supplementary material for: Sleep deprivation alters the time course but not magnitude of locomotor sensitization to cocaine
Source: Sci Rep. 2018 Dec 5;8:17672. doi: 10.1038/s41598-018-36002-1 (PMC6281608; doi:10.1038/s41598-018-36002-1)
Supplement: Supplementary file 1 — Supplementary information [file 41598_2018_36002_MOESM1_ESM.docx]

Title: **Sleep deprivation alters the time course but not magnitude of locomotor sensitization to cocaine.**

Theresa E. Bjorness* ^a,b^

Robert W. Greene ^b,c,d,e^

^a^Research Service, North Texas VA Health Care System, Dallas, TX 75216

^b^Department of Psychiatry, University of Texas Southwestern, Dallas, TX 75390

^c^Department of Neuroscience, University of Texas Southwestern, Dallas, TX 75390

^d^Research Service, North Texas VA Health Care System, Dallas, TX, 75216

^e^International Institute for Integrative Sleep Medicine, University of Tsukuba, Tsukuba, Japan 305-8577

Supplementary Methods: Using the same data set described in the main text, percent change from the previous day (last acclimation day through challenge) was compared using a Two-way repeated measures ANOVA with Tukey corrections for multiple comparisons.

Supplementary Results: The retention of similar sensitization despite hypoactivity in response to the initial cocaine administration was due to a large increase in activity on the second cocaine treatment day in the Coc+SD group compared to the Coc and SD groups (Two-way ANOVA, day *p<0.0001,* group *p=0.0013,* day*group interaction *p<0.0001,* specific group differences via Tukey’s multiple comparison test provided in figure, Figure S1).

Figure S1: Time course of increases in activity across days as determined by percent change from the previous day. Both Coc and Coc+SD groups show large increases in activity on the first treatment day (T1) compared to acclimation (A); however, this increase is significantly higher in the Coc group. Additionally, the Coc+SD group also showed significantly greater activity increases on the first to second treatment day (T1 T2) compared to the Coc and SD groups. All values are expressed as mean+/-SEM, n=10-13/group, ***p<0.005*, ****p<0.0005*, *****p<0.0001* and bars denote which groups differ.


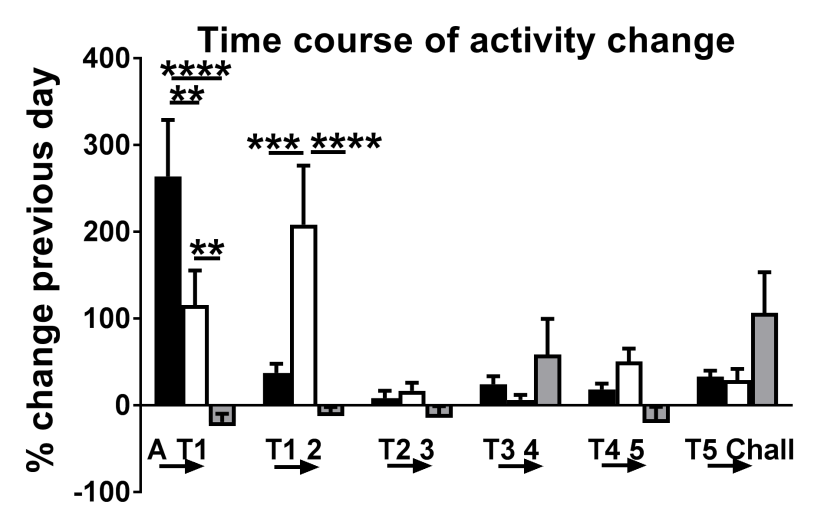


Supplementary Information (data set not described in manuscript): EEG and EMG activity was collected from 4 male C57BL/6 mice across multiple days of acute 4 h sleep deprivation as part of a previously published experiment describing adenosinergic control of homeostatic sleep need[^1^](#_ENREF_1), although sleep rebound, as determined by the increase in slow wave activity power during slow wave sleep (SWS SWA), across repeated deprivations was not previously analyzed.

Supplementary Methods (see[^1^](#_ENREF_1) for full description): Briefly, mice were implanted with EEG and EMG electrodes under isoflurane anesthesia, allowed to recover, acclimated to the recording tethers and the treadmill apparatus after which EEG and EMG activity was measured for 2 days of baseline (undisturbed) and 7 days of 4 h acute sleep deprivation by the treadmill method. EEG/EMG activity was scored offline using a custom Matlab sleepscorer module using standard sleep staging criteria. SWS SWA was averaged in 1 h bins and rebound was calculated as the change in SWS SWA following sleep deprivation compared to the same circadian time under baseline conditions. Rebound was compared for the first 4 h following sleep deprivation on the basis that SWS SWA returned to baseline levels within this period on all days (as determined by a One sample t test with a comparison to a theoretical mean of 0).

Supplementary Results: There was no difference in average rebound SWS SWA within the first 4 h following sleep deprivation across 7 days of 4 h acute sleep deprivation (data not shown, One-way repeated measures ANOVA, *p=0.2*), while the time course of decline in rebound following sleep deprivation was similar on the first, middle, and last days (Two-way ANOVA repeated measures ANOVA, time *p=0.0011,* day *p=0.87,* time*day interaction *p=0.055*, Figure S2).

Figure S2: SWS SWA rebound does not habituate to repeated acute sleep deprivation. Time course of recovery relative to baseline levels of SWS SWA is shown for the first (SD1), middle (SD4), and last (SD7) 4 h sleep deprivation days. All values are expressed as mean+/-SEM, n=4.

Supplementary Figure S2:


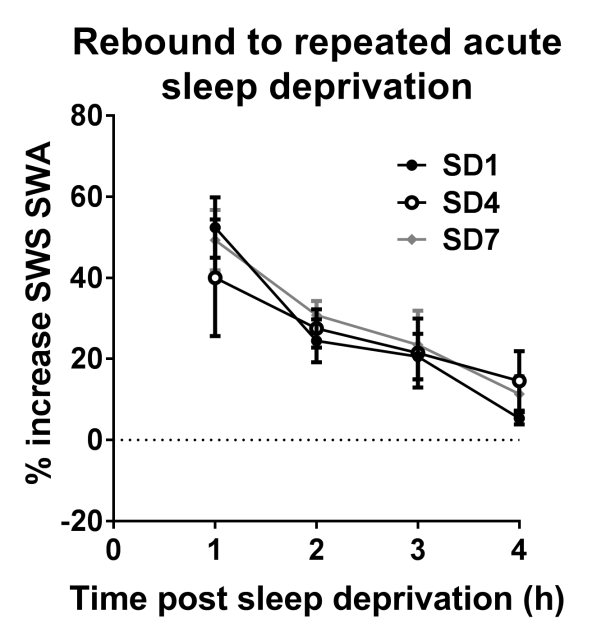


1 Bjorness, T. E. *et al.* An Adenosine-Mediated Glial-Neuronal Circuit for Homeostatic Sleep. *J. Neurosci.* **36**, 3709-3721, doi:10.1523/JNEUROSCI.3906-15.2016 (2016).
